# Microbe Profile: *Aspergillus fumigatus:* a saprotrophic and opportunistic fungal pathogen

**DOI:** 10.1099/mic.0.000651

**Published:** 2018-08-01

**Authors:** Wenxia Fang, Jean-Paul Latgé

**Affiliations:** ^1^​ National Engineering Research Center for Non-Food Biorefinery, State Key Laboratory of Non-Food Biomass and Enzyme Technology, Guangxi Key Laboratory of Bio-refinery, Guangxi Academy of Sciences, Nanning, PR China; ^2^​ Unité des Aspergillus, Institut Pasteur, Paris, France

**Keywords:** *Aspergillus fumigatus*, cell wall, Immune response, saprotrophic, polysaccharides

## Abstract

*Aspergillus fumigatus* is a saprotrophic fungus that continuously disseminates spores (conidia) into the environment. It is also the most common and opportunistic aerial fungal pathogen, causing allergic and chronic lung pathologies including the fatal invasive aspergillosis in immunocompromised patients. The pathobiology of aspergillosis is complex and depends on the competence of the host immune system. Moreover, *A. fumigatus* has become a model to study unique features of fungi. This includes the fungal cell wall, which not only acts as a rigid skeleton for protection against hostile environments but also plays significant roles during infection by manipulating the host immune response.

## Taxonomy

Kingdom *Fungi*, phylum *Ascomycota*, class *Eurotiomycetes*, order *Eurotiales*, family *Aspergillaceae*, genus *Aspergillus*, and species *fumigatus*.

## Properties

*A. fumigatus* is a ubiquitous saprotrophic fungus that plays an important role in recycling carbon and nitrogen on earth but can also be a lethal opportunistic lung pathogen (see graphical abstract). It is a trimorphic fungus with vegetative mycelium that contributes to decay of organic materials in soil, asexual conidia responsible for aerial dispersal of the species and dormant ascospores that ensure the organism’s long-term survival [1]. Additionally, the cell wall of *A. fumigatus* has unique features which allow the fungus to survive under antagonistic environments but also play a major role in the interactions with the mammalian immune system.

## Genome

The first sequenced and most commonly used genomic reference for *A. fumigatus* is the clinical isolate Af293, which consists of eight chromosomes with a stable haploid genome of 29.4 megabases, encoding 9926 predicted genes [2]. The second sequenced genome was the clinical strain A1163, which apart from 2 % of unique genes, has almost identical core genes to Af293 [3]. To date, twelve genomes of various *A. fumigatus* strains have been sequenced. However, only Af293 has been completely assembled at the chromosome level, while A1163 and six other strains have been assembled and released as scaffolds and the remaining four have been released as contigs. Interestingly, large-scale genome comparisons have revealed extensive enzymatic machinery that is mainly acquired to degrade the plant cell wall, such as the strain Z5 isolated from compost possessing large numbers of cellulases, hemicellulases and pectinases that are involved in lignocellulosic biomass degradation [4]. Conversely, despite its ability to infect mammalian hosts no unique gene sets are shared with other human fungal pathogens [5]. Together, this genomic information suggests that the primary ecological niche of *A. fumigatus* is the plant [5].

## Phylogeny

The genus *Aspergillus* contains over 300 species. *A. fumigatus* is the most abundant in the environment and the major cause of invasive aspergillosis (IA), followed by *A. lentulus*, *A. viridinutans, A. udagavae, and A. thermomutatus.* These strains often display comparable levels of intrinsic resistance to antifungal drugs, making it difficult to distinguish between strains using standard morphological and sequence analyses. Therefore, in the case of IA infection, it is necessary to combine morphological examination with other characteristic profiling, multiple PCR and MALDI-TOF techniques to diagnose and improve disease management [6].

## Key Features and Discoveries

In 1729, Italian priest Pier Antonio Micheli named the mold *Aspergillus* in reference to the shape of a holy water sprinkler; while the species name *fumigatus* comes from George W. Fresenius in 1863, referring to the ‘smoky clouds’ of blue-grey conidia. *A. fumigatus* optimally grows at 37 °C (ranging from 15–55 °C) but can survive at 70 °C. Being a saprotroph, *A. fumigatus* can grow in many nutritional environments to produce vegetative mycelia. However, upon starvation dormant conidia (2–3 µm in diameter) are produced that will only germinate in favorable environments.

*Aspergillus* species are known to cause infection in humans and animals with a range of manifestations from localised infections to fatal disseminated diseases [7]. The first recorded *Aspergillus* human infection was Jacques Thibault, a French Revolutionary soldier, who suffered from persistent sinusitis. Aspergillosis cases have continuously risen since this diagnosis. Milder, allergic forms of aspergillosis such as Allegic Bronchopulmonary Aspergillosis (ABPA) and aspergilloma are more common than IA. Even so, IA cases worldwide are estimated to be >200 000 annually with a 50 % mortality rate even when diagnosed and treated early [8]. The number of IA cases are even considered underestimated due to poor diagnostic techniques and the very high mortality rate [8].

*A. fumigatus* has several unique biological characteristics which make it the most prevalent pathogen of the genus *Aspergillus*. These include: (1) its ability to disperse high concentrations of conidia into the environment; (2) its thermotolerance and rapid growth rate in adverse environments; (3) its small conidia size that effectively penetrates deep into the respiratory tract; (4) its capacity to acquire nutrients in the limited lung environment using extracellular enzymes [9]; (5) its unique cell wall structure for protection from the host immune system, such as conidia melanin and rodlet layers that block the NADPH oxidase complex [10] and hyphae galactosaminogalactan that masks specific molecular patterns [11]; (6) its secondary metabolites such as gliotoxin and fumagillin that are antagonistic of the host response [12]. These characteristics make *A. fumigatus* a model for studying fungal-host interactions [13].

## Open Questions

- Increase our understanding of the fungal cell wall structure and how it is assembled. Although it is known that the cell wall is basically composed of fibrillar β-1, 3-glucan and chitin embedded in an amorphous cement made of α-1, 3-glucan, galactomannan and galactosaminogalactan, the kinetics of deposition and remodelling of these components as well as their three-dimensional organisation remains poorly understood.
- Integrate microbiology and immunology research better to identify host pathogen recognition receptors/pathogen-associated molecular pattern interactions and unique features for the survival of the fungus in the host.
- Provide increased understanding of the contribution of host microbiota in the establishment of *Aspergillus* infection.
- Understand the process of quiescence of conidia (unable to germinate in water) and the dormancy of ascospores (unable to germinate without a 65 °C heat shock).
- Characterise the nutritional virulence switch of *Aspergillus.* Develop experiments to understand how the host environment contributes to this saprotrophic microorganism’s pathogenic lifestyle.
